# Clinical Remission Criteria and Serum Levels of Type 2 Inflammation Mediators during 24 Weeks of Treatment with the Anti-IL-5 Drug Mepolizumab in Patients with T2-High Severe Asthma

**DOI:** 10.3390/diagnostics14131345

**Published:** 2024-06-25

**Authors:** Jolita Palacionyte, Andrius Januskevicius, Egle Vasyle, Airidas Rimkunas, Skaidrius Miliauskas, Kestutis Malakauskas

**Affiliations:** 1 Department of Pulmonology, Lithuanian University of Health Sciences, LT-44307 Kaunas, Lithuania; skaidrius.miliauskas@lsmu.lt (S.M.); kestutis.malakauskas@lsmu.lt (K.M.); 2 Laboratory of Pulmonology, Department of Pulmonology, Lithuanian University of Health Sciences, LT-44307 Kaunas, Lithuania; andrius.januskevicius@lsmu.lt (A.J.); egle.jurkeviciute@lsmu.lt (E.V.); airidas.rimkunas@lsmu.lt (A.R.)

**Keywords:** T2-high severe asthma, eosinophils, clinical remission, type 2 inflammation mediators, mepolizumab

## Abstract

Anti-interleukin (IL) 5 is an effective treatment modality for inhibiting eosinophilic inflammation in patients with T2-high severe asthma. The aim of this study was to determine the clinical efficacy and serum levels of type 2 inflammatory mediators during 24 weeks of mepolizumab treatment in patients with T2-high severe asthma. Eighteen patients with T2-high severe asthma were enrolled in this study. All patients received 100 mg of mepolizumab subcutaneously every 4 weeks and were retested at 4, 12, and 24 weeks. A clinical examination, asthma control test (ACT), and spirometry were performed; fractional exhaled nitric oxide (Fe_NO_) levels were evaluated; and blood samples were drawn at every visit. Type 2 inflammation mediator levels were measured using enzyme-linked immunosorbent assay (ELISA). The blood eosinophil level significantly decreased, the ACT score and FEV_1_ increased after 4 weeks of mepolizumab treatment with the same tendency after 12 and 24 weeks (*p* < 0.05), and the Fe_NO_ level did not change (*p* > 0.05). A total of 27.8% of patients reached clinical remission criteria after 24 weeks of mepolizumab treatment. IL-33 and eotaxin significantly increased (*p* < 0.05) while IL-5, IL-13, thymic stromal lymphopoietin (TSLP), soluble IL-5 receptor subunit alpha (sIL-5Rα), and soluble high-affinity immunoglobulin E receptor (sFcεRI) decreased, with the same tendency after 12 and 24 weeks (*p* < 0.05). The serum levels of immunoglobulin (Ig) E and IL-4 and IL-25 levels did not change during mepolizumab treatment compared to baseline (*p* > 0.05). In conclusion, treatment with mepolizumab over 24 weeks improved lung function and asthma control in T2-high severe asthma patients, with nearly one-third achieving clinical remission criteria, and affected the balance of type 2 inflammatory mediators.

## 1. Introduction

Eosinophilic asthma is an asthma phenotype characterized by eosinophilic airway inflammation [1]. This phenotype is predominant in approximately half of asthma patients [2]. During eosinophilic airway inflammation, when the airways come into contact with irritants, viruses, and microbes, the epithelial cells release cytokines that activate inflammatory cells and initiate an inflammatory response and eosinophilopoiesis [3]. The role of interleukin (IL)-5 is highly significant as it is one of the main cytokines that affect eosinophils [4]. Eosinophils play a critical role in supporting the inflammatory process by synthesizing a variety of mediators. Increased eosinophil levels in the airways and blood are associated with disease severity [5]. Eosinophilic airway inflammation eventually leads to smooth muscle contraction and structural changes in the airway, leading to reduced lung function and repeated exacerbations [6,7].

The goal of asthma treatment is to achieve good control of symptoms and minimize the future risk of asthma-related mortality, exacerbations, persistent airflow limitation, and side effects of treatment. However, attention has increasingly turned to clinical remission as a treatment goal [8]. Clinical remission is characterized by a high level of disease control, including the absence of asthma symptoms, no exacerbations, and no need for systemic corticosteroids to treat exacerbations, as well as optimal lung function. The possibility of achieving clinical remission appeared more than a decade ago with the development of asthma treatment and the advent of biological medications [9].

Monoclonal antibodies directed against type 2 inflammatory cytokines and their receptors effectively treat T2-high severe asthma [10]. IL-5 is often selected as a priority target for reducing eosinophilia due to its broad effects on eosinophil physiology [11]. Blocking IL-5 prevents it from binding to receptors on the surface of eosinophils, thus inhibiting the biological activity of this cytokine [12]. Our hypothesis for this study is that anti-IL-5-directed treatment affects not only IL-5 but also other inflammation mediators. We chose to study epithelium-derived alarmin cytokines, which play a crucial role in the initiation of inflammation; the main type 2 inflammation cytokines, which promote airway eosinophilia; eotaxins, which stimulate the migration of eosinophils; and soluble receptors involved in the pathogenesis of type 2 inflammation.

The aim of this study was to determine the clinical efficacy and serum levels of type 2 inflammatory mediators during 24 weeks of mepolizumab treatment in patients with SEA.

## 2. Materials and Methods

### 2.1. Study Design

The participants in this study were recruited from the Department of Pulmonology at the Hospital of Lithuanian University Health Sciences Kaunas Clinics. Eligible patients were selected as they arrived at the outpatient clinic for a T2-high severe asthma treatment consultation. This study lasted from December 2020 to August 2023. Table 1 shows the inclusion and exclusion criteria for subject participation.

**Figure 1:**
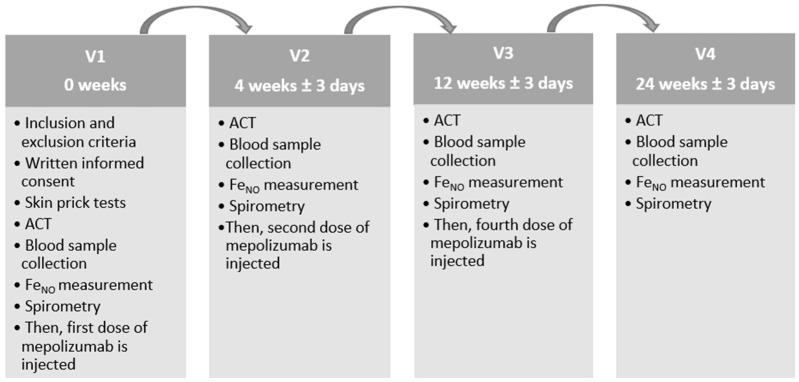
Research design. ACT, asthma control test; Fe_NO_, fractional exhaled nitric oxide; V1, visit before starting mepolizumab treatment; V2, visit 4 weeks after starting mepolizumab treatment (after 1 mepolizumab dose); V3, visit 12 weeks after starting mepolizumab treatment (after 3 mepolizumab doses); V4, visit 24 weeks after starting mepolizumab treatment (after 6 mepolizumab doses).

Once the inclusion and exclusion criteria were approved, all eligible applicants were invited to the study no later than four weeks afterward. Before the initiation of research, each subject was given ample time to review, comprehend, and sign the informed consent. Once all subjects signed the informed consent form, the investigation began. After arrival at the hospital, participants were required to stay 30–60 min. All participants visited the hospital four times: before starting mepolizumab treatment (V1), 4 weeks after starting treatment (after 1 dose of mepolizumab, V2), 12 weeks after starting treatment (after 3 doses of mepolizumab, V3), and 24 weeks after starting treatment (after 6 doses of mepolizumab, V4). Figure 1 depicts the research design.

In this analysis, we used a four-component clinical remission definition that required patients to meet all these criteria for the following 24 weeks: short-term (≤14 days) oral corticosteroid free; exacerbation free; an asthma control test (ACT) score of ≥20; and a percent predicted post-bronchodilator forced expiratory volume in 1 s (FEV_1_) of ≥80% [13].

### 2.2. Pulmonary Function Tests

#### 2.2.1. Spirometry

Spirometry was carried out on all participants at least three times. Of the results collected, the highest of the three independent measurements was considered. An ultrasonic spirometer was utilized for lung function testing (Ganshorn Medizin Electronic, Niederlauer, Germany). The description of this performance, including the procedure used and technique details, can be found in [14].

#### 2.2.2. Fractional Exhaled Nitric Oxide (Fe_NO_) Test

Fe_NO_ analysis was performed during every encounter for all participants involved in this study. A handheld Vivatomo-me device (Bosch Healthcare Solutions, Waiblingen, Germany) was used for the aforementioned Fe_NO_ analysis, with measurements produced according to the manufacturer’s instructions. The details and techniques employed for the performance of this procedure are described in [15].

### 2.3. Blood Tests

#### 2.3.1. Complete Blood Count and IgE

Peripheral blood samples were collected from all participants and added to vacutainers containing dipotassium ethylenediaminetetraacetic acid (K2EDTA) (BD Vacutainer^®^, Becton Dickinson UK Ltd., Wokingham, UK). After collection, the routine clinical chemistry assay samples were promptly sent to the hospital laboratory. An XE-5000 (Sysmex, Kobe, Japan) and a UniCel^®^ DxH 800 Coulter^®^ Cellular Analysis System automated hematology analyzer (Beckman Coulter, Miami, FL, USA) were employed for the complete blood count test. An AIA-2000 automated immunoassay analyzer (Tosoh Bioscience, South San Francisco, CA, USA) was employed for the immunoglobulin (Ig) E test. During processing, the samples were rigorously controlled and monitored. More detail on the description technique used can be found in [15].

#### 2.3.2. Measurement of Blood Levels of Selected Analytes

##### Enzyme-Linked Immunosorbent Assay (ELISA)

Most biomarkers in blood samples were detected using an ELISA. All assays were performed according to the manufacturer’s instructions. Firstly, blood serum and standard curve samples were added to the assay plates pre-coated with analyte-specific capture antibodies. After incubation and washing steps, antibody–horseradish peroxidase conjugates specific to the analytes of interest were added to form an antibody–antigen sandwich. Finally, the HRP substrate was added. After incubation, the reaction was stopped, and the color intensity was measured with a spectrophotometer at a wavelength of 450 nm. The investigated biomarker concentrations were calculated from standard curves. The sensitivity for ELISA kits was as follows: IL-4, 1.3 pg/mL; IL-5, 1.45 pg/mL; IL-13, 0.7 pg/mL; IL-25, 0.4 ng/mL; IL-33, 0.9 pg/mL; thymic stromal lymphopoietin (TSLP), 3 pg/mL; soluble IL-5 receptor subunit alpha (sIL-5Rα), 150 pg/mL; eotaxin, 2.2 pg/mL; and soluble high-affinity IgE receptor (sFcεRI), 68 pg/mL.

### 2.4. Skin Prick Testing

The subjects’ sensitization to allergens was evaluated by performing skin prick tests with *Dermatophagoides pteronyssinus*, *Dermatophagoides farinae*, dog and cat dander, five mixed grass pollens, birch pollen, mugwort, *Alternaria*, *Aspergillus*, and *Cladosporium*. This testing was performed using standardized allergen extracts (Stallergenes, S.A., Antony, France).

### 2.5. Confirmation of Participation in the Study

This study was initiated after obtaining prior approval from the bioethics committee (BE-2-58, 19 June 2020). All subjects were required to sign an informed consent form before this study began. A sufficient amount of time was allotted for subjects to become familiar with the aspects of the study protocol, wherein all of their relevant questions were addressed. After the subjects signed the protocol, investigations were initiated. In the interest of anonymization, unique numbers were allocated for all data. The subject’s safety, dignity, and well-being were ensured throughout the investigation; the interests of the subjects were considered more important than all other interests. Registration of this study with the identification number NCT04542902 was submitted on ClinicalTrial.gov.

### 2.6. Statistical Analysis

Statistical analysis was performed utilizing SPSS statistical software (IBM SPSS Statistics 20; Chicago, IL, USA). The Shapiro–Wilk test was applied to assess the assumption of normality in data distribution. It was found that the data distribution did not pass the normality test; therefore, the nonparametric Mann–Whitney two-sided U-test and Wilcoxon matched-pairs signed-rank two-sided test were applied. It is noted that the threshold value for statistically significant differences was *p* < 0.05.

## 3. Results

### 3.1. Study Subject Characteristics

A total of 18 subjects aged from 37 to 80 years old with T2-high severe asthma participated in the study. Few patients self-reported chronic sinusitis with nasal polyps and none reported eosinophilic granulomatosis with polyangiitis or eosinophilic pneumonia. We examined patients at four timepoints: before starting treatment with a 100 mg subcutaneous injection of mepolizumab every 4 weeks (V1) and 4 (V2), 12 (V3), and 24 (V4) weeks after starting mepolizumab treatment.

Most study subjects were women (77.8%) without allergies (55.6%) and tended to be overweight. Before add-on treatment with a 100 mg subcutaneous injection of mepolizumab, 100% of patients had a recurrent exacerbation. After the first dose of mepolizumab, no asthma exacerbations were observed; however, after the second dose of mepolizumab, one patient experienced an asthma exacerbation. Subsequent continuation of mepolizumab treatment did not increase the number of patients experiencing asthma exacerbations.

**Figure 2:**
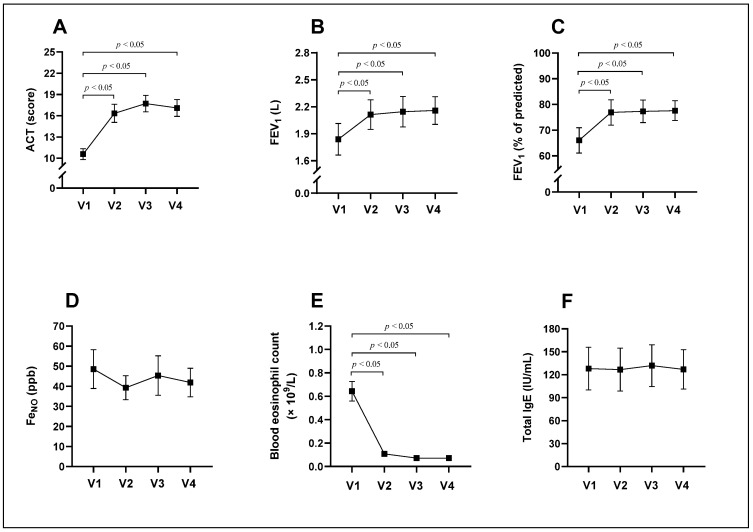
ACT scores (**A**), FEV_1_ (**B**,**C**), Fe_NO_ (**D**), blood eosinophil count (**E**), and total IgE (**F**) levels during 24 weeks of treatment with the anti-IL-5 drug mepolizumab. ACT, asthma control test; Fe_NO_, fractional exhaled nitric oxide; FEV_1_, forced expiratory volume in 1 s; Ig, immunoglobulin; V1, visit before starting mepolizumab treatment; V2, visit 4 weeks after starting mepolizumab treatment (after 1 mepolizumab dose); V3, visit 12 weeks after starting mepolizumab treatment (after 3 mepolizumab doses); V4, visit 24 weeks after starting mepolizumab treatment (after 6 mepolizumab doses). Data presented as the mean ± standard error of the mean. Statistical analysis—Wilcoxon matched-pairs signed-rank test.

Four weeks after starting treatment with mepolizumab, the ACT and FEV_1_ significantly increased; however, the blood eosinophil level significantly decreased, an effect that remained throughout the treatment (*p* < 0.05) (Table 2, Figure 2A–C,E).

The Fe_NO_ level was not significantly different either before or during treatment (*p* > 0.05). It should be noted that for several times, this measurement result was not registered because the patient was unable to control the strength of the breath during the measurement despite the control visualization help on the device screen. The IgE level was also not significantly different before or during treatment (*p* > 0.05) (Table 2, Figure 2D,F).

### 3.2. Severe Asthma Clinical Remissions Criteria at 24 Weeks of Mepolizumab Treatment

**Figure 3:**
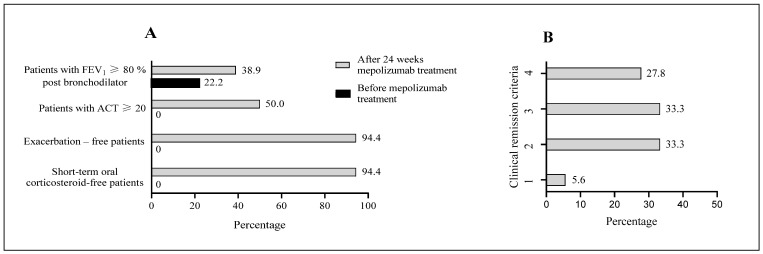
Number of patients meeting individual criteria for clinical remission in asthma after 24 weeks of mepolizumab treatment (**A**). Number of patients meeting one, two, three, or four criteria for clinical remission following 24 weeks of mepolizumab treatment (**B**). ACT, asthma control test; FEV_1_, forced expiratory volume in 1 s.

In the 18 patients analyzed, after 24 weeks of mepolizumab treatment, the number of exacerbation-free patients and short-term systemic corticosteroid-free patients increased from 0 (0%) to 17 (94.4%), the number of patients with an ACT score of ≥20 increased from 0 (0%) to 9 (50%), and the number of patients with an FEV_1_ of ≥80% post-bronchodilator increased from 4 (22.2%) to 7 (38.9%) (Figure 3A). After 24 weeks of treatment with mepolizumab, five patients (27.8%) fulfilled the four-component clinical remission definition, and six patients (33.3%) fulfilled the three-component definition. Before starting mepolizumab treatment, zero patients (0%) met these criteria. Moreover, after 24 weeks of treatment with mepolizumab, six patients (33.3%) fulfilled the two-component clinical remission definition, and one patient (5.6%) fulfilled the one-component definition. Before starting mepolizumab treatment, four and zero patients (0%; 22.2%), respectively, met these criteria (Figure 3B).

### 3.3. Serum Levels of Type 2 Inflammation Mediators during 24 Weeks of Treatment with the Anti-IL-5 Drug Mepolizumab

**Figure 4:**
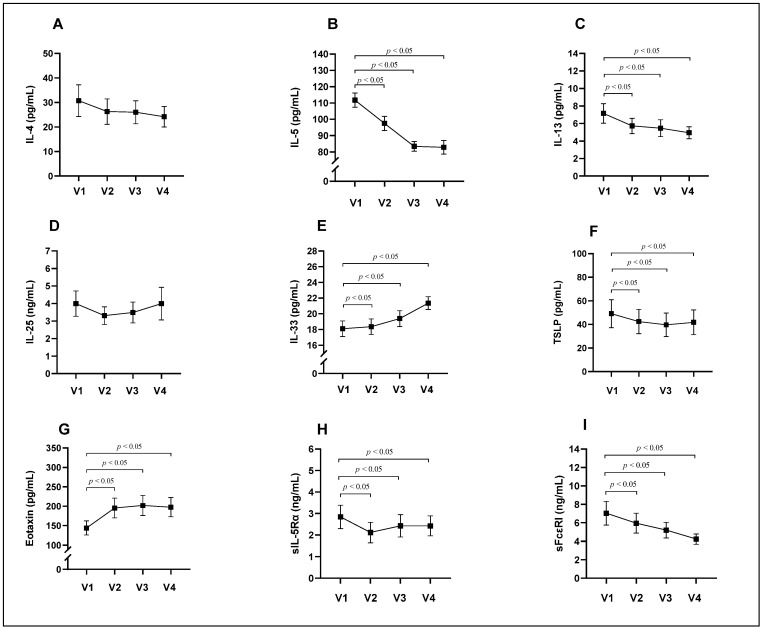
Serum levels of IL-4 (**A**), IL-5 (**B**), IL-13 (**C**), IL-25 (**D**), IL-33 (**E**), TSLP (**F**), eotaxin (**G**), sIL-5Rα (**H**), sFcεRI (**I**) during 24 weeks of treatment with the anti-IL-5 drug mepolizumab. IL, interleukin; sFcεRI, soluble high-affinity IgE receptor; sIL-5Rα, soluble IL-5 receptor subunit alpha; TSLP, thymic stromal lymphopoietin; V1, visit before starting mepolizumab treatment; V2, visit 4 weeks after starting mepolizumab treatment (after 1 mepolizumab dose); V3, visit 12 weeks after starting mepolizumab treatment (after 3 mepolizumab doses); V4, visit 24 weeks after starting mepolizumab treatment (after 6 mepolizumab doses). Data presented as the mean ± standard error of the mean. Statistical analysis—Wilcoxon matched-pairs signed-rank test.

This study found that after 4 weeks of mepolizumab treatment, the serum levels of IL-4 and IL-25 significantly did not change (*p* > 0.05) (Table 3, Figure 4A,D). Meanwhile, the serum levels of IL-5, IL-13, TSLP, sIL-5Rα, and sFcεRI significantly decreased (*p* < 0.05), and this effect remained throughout the treatment (Table 3, Figure 4B,C,H,I). The serum levels of IL-33 and eotaxin significantly increased considerably after 4 weeks of mepolizumab treatment (*p* < 0.05), and this effect remained throughout the treatment (Table 3, Figure 4E,G).

## 4. Discussion

In this study, an initial 100 mg dose of mepolizumab administered subcutaneously in patients with T2-high severe asthma significantly increased ACT scores and improved lung function, and this effect was sustained throughout all 24 weeks of treatment. Almost one-third of the subjects reached clinical remission criteria after 24 weeks of mepolizumab treatment. This study highlights the effects of mepolizumab treatment on various serum type 2 mediators in patients with T2-high severe asthma. It was observed that after 4 weeks of treatment with mepolizumab, the serum levels of IL-5, IL-13, TSLP, sIL-5Rα, and sFcεRI experienced a significant decrease that persisted throughout the treatment period. In contrast, the levels of IL-4 and IL-25 remained unchanged. Conversely, the serum levels of IL-33 and eotaxin showed a notable increase from the beginning of the treatment, a trend that continued over time. This indicates a specific and targeted effect of mepolizumab on certain type 2 inflammatory markers and immune responses.

In this study, a significant increase in ACT scores was observed. These results demonstrate the severity of disease in T2-high severe asthma despite receiving standard-of-care treatment in line with the Global Initiative for Asthma (GINA) recommendations up to the initiation of biologic therapy. This study demonstrated that mepolizumab elicited long-lasting improvements in lung function (increased FEV_1_) and eosinophilic inflammation attenuation (decreased eosinophils level). This effect is closely related to the mepolizumab-mediated improvement in asthma control. The results of our study are consistent with those of other studies [16,17,18,19,20]. In this study, we found that mepolizumab did not change IgE levels, which is in agreeance with the results of other studies [21,22]. This is because mepolizumab blocks IL-5 and may not affect other components of type 2 inflammation, such as IgE. Fe_NO_ also remained largely unchanged during treatment with mepolizumab, which is consistent with findings from other studies [20,23]; in contrast, one study observed a significant increase in the Fe_NO_ level [24]. We hypothesize that although mepolizumab blocks the IL-5 pathway and thus inhibits type 2 inflammation, the Fe_NO_ levels are not significantly altered because this biomarker is not directly related to the IL-5 pathway. Note that all inflammatory pathways are interrelated. It is possible that blocking the IL-5 upregulates IL-13 and IL-4 pathways, which increases Fe_NO_ through compensatory mechanisms.

Asthma remission is defined as a sustained absence of signs and symptoms and normalizing and optimizing lung function. The biologics achieve multiple criteria for asthma remission on treatment [25]. Our results showed that after 24 weeks of treatment with mepolizumab, almost two-thirds of patients met three or four criteria for asthma remission. We also evaluated each criterion separately before and after 24 weeks of mepolizumab treatment. No patient before mepolizumab treatment did not meet the following criteria: corticosteroid-free, exacerbation-free, and with an ACT score of ≥20. After 24 weeks of mepolizumab treatment, these results significantly improved. Many studies have shown that mepolizumab reduces the number of asthma exacerbations, improves lung function and asthma control, and reduces the frequency of systemic corticosteroid use [26,27,28,29]. The results of our study are consistent with those of previously published studies. We believe that patients have been appropriately selected and that the treatment effectively inhibits eosinophilic inflammation.

IL-5 is one of the most important cytokines in type 2 asthma pathogenesis. This cytokine is involved in the recruitment, activation, and survival of eosinophils. IL-5 inhibition reduces eosinophilic airway inflammation [25]. Although treatment effectively decreases eosinophil numbers, thereby alleviating asthma-related manifestations, it does not render the patients completely cured. It is not known how anti-IL-5 treatment affects the blood levels of pro-inflammatory biological active substances and how organisms react to a reduction in eosinophils. Instead, organisms’ homeostatic mechanisms may compensate by modulating the levels of various blood cytokines. Specifically, it is hypothesized that not all cytokine levels will decrease post-treatment. Some proinflammatory cytokines may remain elevated or even increase as the body seeks to maintain a balanced immune response.

The bronchial epithelium secretes IL-25, IL-33, and TSLP. These cytokines initiate intracellular signal transduction and are called alarmins. Alarmins stimulate the release of IL-4, IL-5, and IL-13 and 2-type inflammation development [3,30]. A previous study already found changes in serum levels of alarmins after a single dose of mepolizumab, in patients with severe non-allergic eosinophilic asthma [31]. Although mepolizumab blocks IL-5, our results show that the inhibition of type 2 inflammation reduced the serum TSLP and increased the IL-33 levels. Meanwhile, the IL-25 levels did not change. We can attribute this to the fact that not only do alarmins stimulate the release of cytokines, but there is also a feedback relationship involving the effects of type 2 cytokines on the epithelium. While eosinophils possess receptors for all three alarmins, evaluating their primary functions, only IL-25 and IL-33 are identified as directly acting on eosinophils after binding to IL-17RB and ST2 receptors on eosinophils [32,33,34]. Instead, TSLP’s role is more upstream in the allergic inflammation cascade, where it helps shape the immune environment towards a type 2 response, indirectly leading to eosinophil activation and recruitment through the actions of other cytokines produced by Th2 cells [35]. Despite the targeted reduction in eosinophils through biological therapy, our findings suggest a complex compensatory response within the cytokine network, highlighting the resilience of the immune system’s regulatory mechanisms. Not only do epithelial cells produce IL-25, but eosinophils are also a significant source of this alarmin [36]. Notably, while the direct impact of biological therapy led to expected reductions in eosinophil counts, the anticipated decrease in IL-25 levels was not observed. This discrepancy suggests that other cellular contributors, potentially including the epithelium, may compensate for the reduced eosinophilic production of IL-25, maintaining its serum levels. Such a compensatory mechanism underscores the intricate balance between eosinophil activity and cytokine production, with IL-33 levels rising, possibly as a direct response to the diminished eosinophil count. Furthermore, our analysis extends to the role of TSLP, which diverges from the actions of IL-25 and IL-33 by primarily activating dendritic cells, not eosinophils. The reduction in eosinophil counts appears to attenuate the aggressive nature of dendritic cells, potentially disrupting the TSLP-mediated activation pathway. This observation aligns with discussions on the compensatory mechanisms within the immune system, where a decrease in one cytokine due to targeted therapy does not necessarily lead to a straightforward decrease in associated inflammatory markers due to the complex feedback loops and interdependencies within the immune network.

IL-4 and IL-13 are among the most important type 2 inflammation cytokines [37]. The main cellular sources of IL-4 and IL-13 include T-helper (Th) lymphocytes, T follicular helper (Tfh) cells and type 2 innate lymphoid (ILC2) cells; eosinophils, basophils, mast cells, natural killer cells, and clusters of differentiation 8 (CD8^+^) T lymphocytes also significantly contribute to the production of these two cytokines [38]. While IL-4 is described to be more important in allergic asthma cases, it also plays a significant role in non-allergic phenotypes. Alarmins-activated ILC2 and Th2 cells produce enhanced levels of IL-4 [39]. IL-33 is a particularly important alarmin that affects IL-4 production [40]. In our study, although serum IL-33 levels increased during mepolizumab treatment, there was no significant effect on IL-4 levels. The unchanged IL-4 level in serum during treatment indicates the compensatory mechanisms of IL-4.

IL-4 and IL-13 affect many cells, such as B cells, eosinophils, basophils, monocytes, and fibroblasts [37]. They both share a receptor component with each other and directly contribute to the tissue inflammation and remodeling seen in diseases such as asthma; they also play a significant role in recruiting eosinophils to the site of inflammation. Both cytokines synergize to amplify allergic responses. Differently from IL-4, the IL-13 levels continuously decreased after the inhibition of type 2 inflammation by mepolizumab treatment. While most IL-4-producing cells can express IL-13, a reduced eosinophil count is related to a reduction in IL-13, with no compensatory mechanisms seen with IL-4. This may suggest that IL-13 production is more associated with eosinophilic activity and inflammation as it is the main cytokine produced by Th2 and ILC2 cells and directly targets eosinophils [41].

Both cytokines IL-4 and IL-13 are associated with the induction of eosinophil chemotaxis, which stimulates the production of eotaxin [42]. Eotaxin is a small protein that is synthesized by a number of different cell types (e.g., epithelial cells, smooth muscle cells, and fibroblasts). Eotaxin is stimulated by IL-4 and IL-13, produced by T-helper lymphocytes. Eotaxin stimulates the recruitment of eosinophils from the airway micro vessels into the lung tissue [43]. We detected that the serum’s eotaxin levels significantly increased during mepolizumab treatment. The mepolizumab treatment reduced the number of eosinophils in the blood as well as in the lung tissue [44]. Eosinophils themselves can modulate the production of chemokines, including eotaxins, through direct interactions with other cells or through the cytokines they release. The absence or reduction in such regulatory functions may lead to the overproduction of eotaxins by other cells in the immune system, such as epithelial cells, endothelial cells, and fibroblasts. Furthermore, the body, recognizing the decreased number of available eosinophils, initiates a compensatory mechanism to recruit more eosinophils to the sites where they are typically needed, such as inflamed airways. To achieve this, the body increases the production of eotaxin, a potent chemokine responsible for eosinophil recruitment. This response is a natural attempt by the organism to maintain homeostasis and manage inflammatory processes effectively, especially under the threat or actual presence of inflammation, where eosinophils play a critical defensive role.

We investigated the blood levels of two soluble receptors—IL-5 receptor sIL-5Rα and IgE receptor FcεRI. The IL-5R is a high-affinity receptor expressed on eosinophils, basophils, and mast cells [45]. This receptor complex is intricately composed of two subunits: IL-5Rα and βc. While IL-5Rα uniquely engages with IL-5, facilitating a specific interaction, the βc subunit does not directly bind to IL-5 but is essential for downstream signaling processes [46]. sIL-5Rα emerges through either alternative splicing of the mRNA transcript or proteolytic cleavage of the membrane-bound receptor. This soluble receptor variant retains the ability to bind IL-5, albeit without initiating traditional cell signaling due to its lack of attachment to the cell surface. The production of sIL-5Rα via these mechanisms allows it to function in several ways, such as acting as a decoy receptor that can bind to IL-5, thereby regulating its availability and activity in the extracellular environment. This can modulate the biological effects of IL-5, influencing immune responses and inflammation. We found that the serum levels of soluble IL-5Rα significantly decreased during treatment with mepolizumab. If sIL-5Rα performs a regulatory function for IL-5, after mepolizumab treatment, the organism may attempt to maintain the current IL-5 levels; thus, sIL-5Rα will not be secreted into the blood.

Moreover, we investigated the serum concentrations of the soluble receptor FcεRI, a high-affinity IgE Fc receptor that is expressed on many cell types; eosinophil is also characterized as a cell that expresses this receptor [47,48]. A truncated version of the IgE-binding sFcεRI in human serum [49]. sFcεRI is generally found in two main forms in the blood: the free form and the complexed form, where it is associated with IgE. Although the transmembrane form of FcεRI is primarily expressed by immune cells such as mast cells and basophils, eosinophils have also been suggested as a potential source of the soluble receptor variants, particularly in pathological conditions such as asthma where eosinophil activity is heightened. Our findings indicate that mepolizumab therapy leads to a reduction in circulating levels of sFcεRI. This reduction could be explained by several mechanisms: first, the decrease in eosinophil numbers may directly lower the production of sFcεRI if eosinophils are a contributing source. Second, the reduction in IL-5 activity may lead to a broader downregulation of immune activation, including the pathways involved in sFcεRI production or shedding from the cell surface. Furthermore, with fewer eosinophils and reduced IgE receptor engagement, there may be less cleavage or shedding of the receptor into its soluble form. IgE regulates the expression of high-affinity receptors [50]; moreover, sFcεRI can interfere with IgE detection in serum, which might be of importance in regard to interference in sIgE detection and diagnosis [49]. This nuanced understanding of sFcεRI dynamics offers potential insights into the immunomodulatory effects of IL-5 targeted therapies in asthma, highlighting not only the direct impact on eosinophil viability but also its broader implications for the patient’s immunological landscape. The investigated IL-5 level in patients’ serum during mepolizumab treatment significantly decreased; however, there was still a significant amount of circulating IL-5.

This study has several potential limitations. Firstly, this study had a relatively short observation period spanning only 24 weeks, limiting the evaluation of clinical remission criteria. To assess clinical remission, this research should be continued for 12 months. Secondly, only clinically stable T2-high severe asthma patients free of systemic steroids for at least 1 month before the study were considered; therefore, it remains unclear whether systemic glucocorticoids can affect the level of type 2 inflammatory mediators during treatment with anti-IL-5 mepolizumab. Thirdly, whether patients’ asthma was allergic or non-allergic was not considered.

## 5. Conclusions

The results of this study confirm the efficacy of biologics in the treatment of T2-high severe asthma. Add-on therapy with mepolizumab, an anti-IL-5 treatment, resulted in significant functional and clinical changes. Our study results demonstrate that mepolizumab significantly improved the ACT score and FEV_1_ after the first four weeks of treatment, with the effect persisting during the continuation of treatment. Meanwhile, there was a significant decrease in the number of eosinophils in the blood. Treatment with mepolizumab helped to achieve clinical remission criteria nearly in one-third of the patients with T2-high severe asthma. In addition, the serum levels of type 2 inflammation mediators such as IL-13, IL-33, TSLP, sIL-5Rα, and sFcεRI were significantly altered during treatment. These findings indicate that mepolizumab has a broader impact on T2-high severe asthma pathophysiology.

## Figures and Tables

**Table 1:** Inclusion and exclusion criteria.

Inclusion Criteria	Exclusion Criteria
Severe asthma history ≥ 12 months High doses of inhaled steroids + long-acting beta agonist + episodic use of oral steroids ≥ 12 months Uncontrolled asthma ≥ 12 months Two or more asthma exacerbations per year, which require short-term systemic corticosteroids (≤14 days). Peripheral blood eosinophil ≥ 0.15 × 10^9^/L	Age < 18 years Asthma exacerbation ≤ 1 months prior to study Supported asthma therapy of oral corticosteroid (>30 days) Active airway infection ≤ 1 months prior to study Active smoking (at least one cigarette a day), former smoker (at least 100 cigarettes in lifetime) Clinically significant non-controlled other organs disease

**Table 2:** Demographic and clinical characteristics of the study population.

Characteristic	T2-High Severe Asthma			
Number, n	18			
Sex, M/F	4/14			
Age, years	57.6 ± 2.2			
BMI, kg/m^2^	30.3 ± 1.7			
Number of patients with positive skin prick test/negative skin prick test	8/10			
	V1	V2	V3	V4
Number of patients who had asthma exacerbations	18	0	1	0
ACT, score	10.6 ± 0.8 *^#£^	16.3 ± 1.3	17.7 ± 1.2	17.1 ± 1.2
FEV_1_, L	1.84 ± 0.18 *^#£^	2.11 ± 0.16	2.15 ± 0.17	2.16 ± 0.15
FEV_1_, % of predicted	66.0 ± 4.9 *^#£^	76.9 ± 4.9	77.3 ± 4.4	77.6 ± 3.8
Fe_NO_, ppb	48.6 ± 9.7	39.3 ± 6.0	45.3 ± 9.8	41.9 ± 7.1
Blood eosinophil level, ×10^9^/L	0.64 ± 0.08 *^#£^	0.11 ± 0.02	0.07 ± 0.01	0.07 ± 0.01
Total IgE, IU/mL	128.1 ± 27.9	126.7 ± 28.0	131.9 ± 27.3	127.0 ± 25.7

ACT, asthma control test; F, female; Fe_NO_, fractional exhaled nitric oxide; FEV_1_, forced expiratory volume in 1s; Ig, immunoglobulin; M, male; V1, visit before starting mepolizumab treatment; V2, visit 4 weeks after starting mepolizumab treatment (after 1 mepolizumab dose); V3, visit 12 weeks after starting mepolizumab treatment (after 3 mepolizumab doses); V4, visit 24 weeks after starting mepolizumab treatment (after 6 mepolizumab doses). Data presented as the mean ± standard error of the mean. Statistical analysis—Wilcoxon matched-pairs signed-rank test. * *p* < 0.05 compared with V2; ^#^ *p* < 0.05 compared with V3; ^£^ *p* < 0.05 compared with V4.

**Table 3:** Serum levels of type 2 inflammation mediators during 24 weeks of treatment with the anti-IL-5 drug mepolizumab.

	T2-High Severe Asthma			
	V1	V2	V3	V4
IL-4 (pg/mL)	30.7 ± 6.5	26.3 ± 2.2	26.0 ± 4.7	24.2 ± 4.2
IL-5 (pg/mL)	111.7 ± 4.4 *^#£^	97.5 ± 4.3	83.5 ± 3.0	82.8 ± 4.1
IL-13 (pg/mL)	7.2 ± 1.1 *^#£^	5.7 ± 0.9	5.5 ± 1.0	4.9 ± 0.8
IL-25 (ng/mL)	4.0 ± 0.7	3.3 ± 0.5	3.5 ± 0.6	4.0 ± 0.9
IL-33 (pg/mL)	18. 1 ± 1.0 *^#£^	18.4 ± 1.0	19.4 ± 1.0	21.3 ± 0.8
TSLP (pg/mL)	49.1 ± 11.9 *^#£^	42.4 ± 10.3	39.7 ± 10.0	41.8 ± 10.5
sIL-5Rα (ng/mL)	2.84 ± 0.55 *^#£^	2.11 ± 0.47	2.43 ± 0.52	2.42 ± 0.47
Eotaxin (pg/mL)	143.9 ± 18.2 *^#£^	195.3 ± 25.5	201.7 ± 25.9	197.5 ± 24.8
sFcεRI (ng/mL)	7.0 ± 1.3 *^#£^	5.9 ± 1.1	5.2 ± 0.9	4.2 ± 0.6

IL, interleukin; sFcεRI, soluble high-affinity IgE receptor; sIL-5Rα, soluble IL-5 receptor subunit alpha; TSLP, thymic stromal lymphopoietin; V1, visit before starting mepolizumab treatment; V2, visit 4 weeks after starting mepolizumab treatment (after 1 mepolizumab dose); V3, visit 12 weeks after starting mepolizumab treatment (after 3 mepolizumab doses); V4, visit 24 weeks after starting mepolizumab treatment (after 6 mepolizumab doses). Data presented as the mean ± standard error of the mean. Statistical analysis—Wilcoxon matched-pairs signed-rank test. * *p* < 0.05 compared with V2; ^#^ *p* < 0.05 compared with V3; ^£^ *p* < 0.05 compared with V4.

## Data Availability

Data are contained within the article.

## Abbreviations

ACT	asthma control test
CD8^+^	cluster of differentiation 8
ELISA	enzyme-linked immunosorbent assay
Fe_NO_	fractional exhaled nitric oxide
FEV_1_	forced expiratory volume in 1 s
GINA	Global Initiative for Asthma
Ig	immunoglobulin
IL	interleukin
ILC2	type 2 innate lymphoid
sFcεRI	soluble high-affinity immunoglobulin E receptor
sIL-5Rα	soluble IL-5 receptor subunit alpha
Tfh	T follicular helper
Th	T-helper
TSLP	thymic stromal lymphopoietin
V1	visit before starting mepolizumab treatment
V2	visit 4 weeks after starting mepolizumab treatment (after 1 mepolizumab dose)
V3	visit 12 weeks after starting mepolizumab treatment (after 3 mepolizumab doses)
V4	visit 24 weeks after starting mepolizumab treatment (after 6 mepolizumab doses)
